# Novel roles for class II Phosphoinositide 3-Kinase C2β in signalling pathways involved in prostate cancer cell invasion

**DOI:** 10.1038/srep23277

**Published:** 2016-03-17

**Authors:** Ioanna Mavrommati, Ouma Cisse, Marco Falasca, Tania Maffucci

**Affiliations:** 1 Queen Mary University of London, Barts and The London School of Medicine and Dentistry, Blizard Institute, Centre for Cell Biology and Cutaneous Research, 4 Newark Street, London E1 2AT, UK; 2 Metabolic Signalling Group, School of Biomedical Sciences, CHIRI Biosciences, Curtin University, Perth, Western Australia 6102, Australia

## Abstract

Phosphoinositide 3-kinases (PI3Ks) regulate several cellular functions such as proliferation, growth, survival and migration. The eight PI3K isoforms are grouped into three classes and the three enzymes belonging to the class II subfamily (PI3K-C2α, β and γ) are the least investigated amongst all PI3Ks. Interest on these isoforms has been recently fuelled by the identification of specific physiological roles for class II PI3Ks and by accumulating evidence indicating their involvement in human diseases. While it is now established that these isoforms can regulate distinct cellular functions compared to other PI3Ks, there is still a limited understanding of the signalling pathways that can be specifically regulated by class II PI3Ks. Here we show that PI3K-C2β regulates mitogen-activated protein kinase kinase (MEK1/2) and extracellular signal-regulated kinase (ERK1/2) activation in prostate cancer (PCa) cells. We further demonstrate that MEK/ERK and PI3K-C2β are required for PCa cell invasion but not proliferation. In addition we show that PI3K-C2β but not MEK/ERK regulates PCa cell migration as well as expression of the transcription factor Slug. These data identify novel signalling pathways specifically regulated by PI3K-C2β and they further identify this enzyme as a key regulator of PCa cell migration and invasion.

Phosphoinositide 3-kinases (PI3Ks), the lipid kinases that catalyse the synthesis of the phosphoinositides phosphatidylinositol 3-phosphate, phosphatidylinositol 3,4-bisphosphate and phosphatidylinositol 3,4,5-trisphosphate [PtdIns(3,4,5)*P*_*3*_], have a well-established role in regulation of several cellular processes, including cell proliferation, growth, survival, migration and metabolism^1^,^2^,^3^. Deregulation of PI3Ks-dependent signals is associated with several diseases including cancer^4^,^5^,^6^,^7^,^8^ and indeed inhibitors of this pathway have been developed as anti-cancer drugs and are currently in clinical trials^9^,^10^,^11^. Unfortunately the use of these inhibitors has also led to the discovery of compensatory mechanisms that reduce their therapeutic efficiency^12^,^13^. As a consequence an increasing interest has emerged to better define the potential contribution of each of the eight mammalian PI3K isoforms to cancer development and progression and ultimately to identify more selective and targeted therapeutic strategies^2^,^14^,^15^. Generation of isoform specific knock-out and knock-in mice and the development of isoform-specific inhibitors have greatly improved our knowledge of the physiological roles and the cellular functions that are specifically regulated by each PI3K^16^,^17^. On the other hand much less is known about specific signalling pathways that can be activated selectively by each PI3K isoform.

The three isoforms grouped into the class II subfamily (PI3K-C2α, β and γ) have been the least investigated amongst all PI3Ks for many years^2^,^18^. Lately interest on these enzymes has progressively increased and indeed recent studies have shed light into their specific physiological roles^17^. Attempt to generate homozygous PI3K-C2α knock-out^19^,^20^,^21^ or kinase-inactivating knock-in^22^ mice resulted in embryo lethality revealing a specific requirement for this enzyme in embryo development. On the other hand both PI3K-C2β^−/−^ 23 and PI3K-C2γ^−/−^ 24 mice were viable supporting the conclusion that the class II isoforms have distinct cellular functions. Further investigation identified a pivotal role for PI3K-C2α in endothelial cells, angiogenesis and vascular barrier function^19^, in regulation of primary cilium function^20^ and, more recently, in regulation of platelets membrane structure, morphology and adhesion^21^,^22^. Investigation of PI3K-C2β^−/−^ mice established that the enzyme is not required for growth, differentiation, barrier function and wound healing in the epidermis^23^. PI3K-C2γ appears to have a specific role in glucose homeostasis regulation since PI3K-C2γ^−/−^ mice develop hyperlipidemia, adiposity and insulin resistance with age or following high fat diet^24^. These studies have all reinforced the conclusion that each class II PI3K isoforms can regulate distinct cellular functions compared to other PI3Ks and have fuelled interest towards a better characterisation of these enzymes. A better understanding of potential contribution of class II PI3Ks to human diseases is also urged by accumulating evidence indicating a role for some of these enzymes in cancer. It has been reported that subsets of tumour and cell lines from small cell lung cancer (SCLC), acute myeloid leukemia, glioblastoma multiforme, medulloblastoma^25^ as well as primary neuroblastoma tumours and cell lines^25^,^26^ are characterised by increased levels of PI3K-C2β. Consistently, gene expression profiling indicated increased expression of PI3K-C2β in acute lymphocytic leukemia, acute myeloid leukemia and glioblastoma^27^,^28^,^29^ and recurrent mutations (12.9%) in *PIK3C2B* have been observed in lung cancer^30^. Furthermore PI3K-C2β has been implicated in cancer cell migration^31^,^32^,^33^ and in neuroblastoma tumourigenesis^34^. Importantly inhibition of PI3K-C2β has been shown to inhibit early stage of neuroblastoma formation^34^ and ovarian cancer metastasis formation^33^ in animal models, supporting the conclusion that this enzyme may represent a novel interesting target in anti-cancer therapy. Despite this evidence there is still a very limited understanding of the signalling pathways that can be specifically regulated by PI3K-C2β.

Here we show that PI3K-C2β regulates mitogen-activated protein kinase kinase (MEK1/2) and extracellular signal-regulated kinase (ERK1/2) activation induced by foetal bovine serum (FBS) or epidermal growth factor (EGF) in prostate cancer (PCa) cell lines. Inhibition of MEK/ERK activation as well as downregulation of PI3K-C2β does not affect cell proliferation while specifically inhibiting cell invasion. We further show that PI3K-C2β regulates FBS-induced PCa cell migration in a mechanism that does not appear to involve MEK/ERK activation. Investigation of additional signalling pathways modulated by PI3K-C2β reveals a role for this enzyme in regulating the expression levels of the transcription factor Slug. These data identify novel signalling pathways specifically regulated by PI3K-C2β and involved in migration and invasion of PCa cells.

## Results

### PI3K-C2β regulates MEK/ERK activation in PCa cells

**Figure 1:**
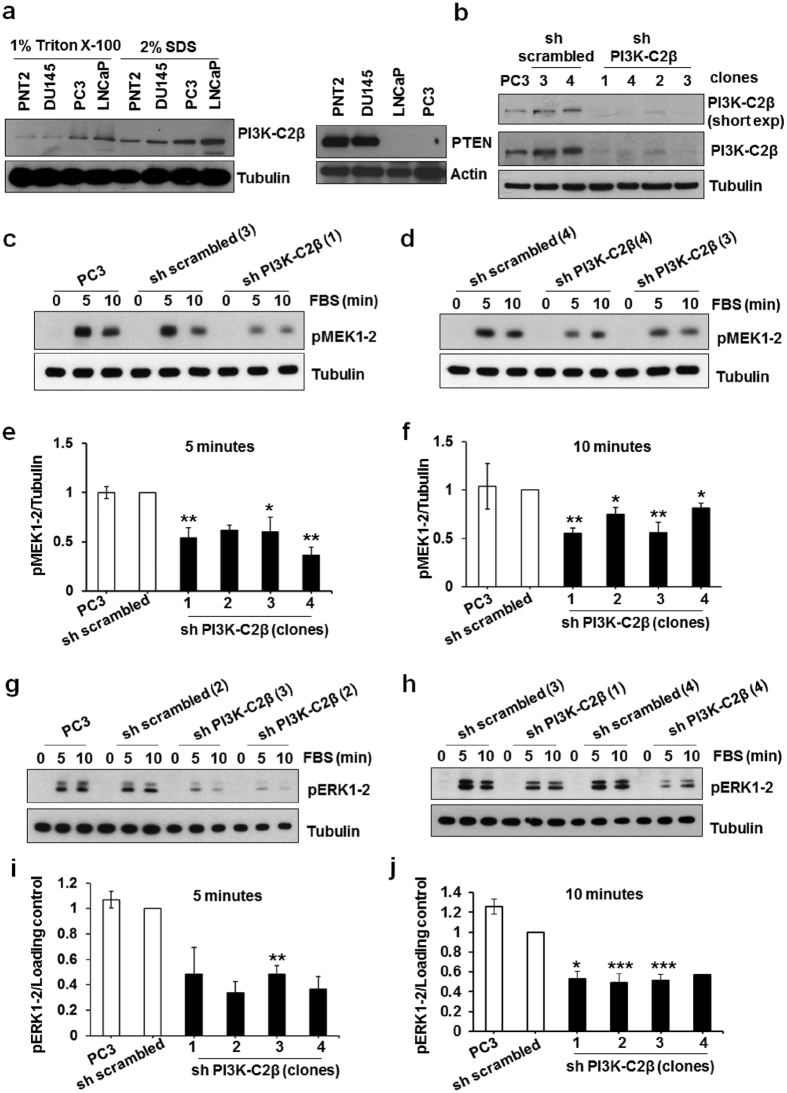
PI3K-C2β is required for FBS-induced MAPK activation. (**a**) Representative blot of PI3K-C2β and PTEN levels in the indicated prostate cell lines upon lysis with 1% Triton X-100 or 2% SDS. Tubulin or Actin were used as loading control. (**b**) Stable PC3 clones expressing a specific shRNA targeting PI3K-C2β or a control shRNA (sh scrambled) were generated as described in the Methods. Representative blots showing expression levels of PI3K-C2β in the indicated stable PC3 cells. Numbers indicate the specific clone. (**c–j**) The indicated PC3 stable clones were serum starved overnight and then stimulated with FBS for the indicated times. Phosphorylation of MEK1/2 at residues Ser217/221 (**c–f**) and ERK1/2 at residues Thr202/Tyr204 (**g–j**) was assessed by Western blotting. Tubulin was used as loading control. In some experiments membranes incubated with pERK1/2 were stripped and then incubated with antibody recognising total ERK2. Representative blots are shown. Results from densitometry analysis are expressed as fold change of the specific sh scrambled clone used in each experiment and are means ± s.e.m. from the following number of experiments: (**e**): n = 3 (PC3 and clone 4), n = 4 (clones 1,3), n = 2 (clone 2); (**f**): n = 3 (PC3, clones 1,4), n = 4 (clone 2), n = 6 (clone 3); (**i**): n = 2 (PC3, clones 1,2,4), n = 3 (clone 3); (**j**): n = 3 (PC3, clone 1), n = 12 (clone 2), n = 11 (clone 3), n = 2 (clone 4). *p < 0.05, **p < 0.01, ***p < 0.001.

The signalling pathways specifically regulated by PI3K-C2β are still not completely defined. While previous studies have mainly focussed their attention on its potential contribution to activation of the well established class I PI3K target Akt^2^,^34^,^35^ and Rho GTPAses^2^,^32^,^36^,^37^ little is known about other kinases potentially regulated by this enzyme. We therefore decided to investigate the potential role of PI3K-C2β on activation of a panel of 43 distinct kinases and 2 related proteins using a phosphokinase antibody array. The choice of the cellular model was prompted by a recent study suggesting a potential association between PI3K-C2β and PCa risk^38^. First we analysed the expression levels of PI3K-C2β in three distinct PCa cell lines compared to PNT2, an immortalised prostate cell line. PI3K-C2β was highly expressed in PC3 and LNCaP cell lines, both lacking the tumour suppressor phosphatase and tensin homolog (PTEN), the phosphatase responsible for dephosphorylation of PtdIns(3,4,5)*P*_*3*_, compared to the PTEN expressing PCa cell line DU145 and to PNT2 cells (Fig. 1a). Interestingly we also noticed that PI3K-C2β was barely detectable in DU145 and PNT2 when cells were lysed with 1% Triton X-100 whereas it was clearly observed when cells were lysed with 2% SDS (Fig. 1a), suggesting a specific localisation of the enzyme within Triton-insoluble cellular fractions in these cells.

Based on PI3K-C2β expression we selected PC3 cells for phosphokinase antibody array analysis. We reasoned that the constitutive hyperactivation of the class I PI3Ks-dependent pathways due to the lack of PTEN could facilitate the identification of pathways specifically modulated by PI3K-C2β upon growth factor stimulation. To determine the role of PI3K-C2β we generated stable PC3 cell lines by transfecting parental cells with a pSuper vector carrying a shRNA specifically targeting the enzyme. Control cells were transfected with a vector containing a non targeting shRNA. Cell populations expressing the PI3K-C2β-targeting shRNA or control shRNA were selected by incubation in media supplemented with puromycin and then allowed to grow as single clones. Four distinct knockdown clones were then selected, all displaying a high efficiency of PI3K-C2β downregulation (sh PI3K-C2β, Fig. 1b, Supplementary Fig. S1a) as well as four control clones (sh scrambled, Fig. 1b, Supplementary Fig. S1a). No effect on the expression levels of the class I isoforms p110α, p110β and the class II enzyme PI3K-C2α was detected in the knockdown clones (Supplementary Fig. S1b,c) and no difference in the expression of PI3K-C2β between control clones and parental cells was observed (Fig. 1b).

Once stable cell lines were established one sh PI3K-C2β clone and one sh scrambled clone were serum starved overnight and either left untreated or stimulated with DMEM containing 10% FBS for 10 min. Activation of a panel of kinases was then analysed in the corresponding lysates (Supplementary Fig. S1d). Densitometry analysis of the phosphoarray indicated only slight differences in the FBS-induced activation of the classical class I PI3K signalling pathways (Supplementary Fig. S1e). On the other hand a clear inhibition of FBS-induced phosphorylation of some members of the mitogen-activated protein kinases (MAPK) family was observed in cells lacking PI3K-C2β (Supplementary Fig. S1f). In particular FBS-induced p38α and MEK1/2 phosphorylation appeared to be strongly reduced in cells lacking PI3K-C2β (Supplementary Fig. S1f).

### Stable PI3K-C2β downregulation inhibits MEK and ERK activation

To validate the results of the array we then performed a time course experiment using two distinct knockdown clones and two control clones (Supplementary Fig. S1g,h). Data revealed a very rapid and transient phosphorylation of ERK1/2 at residues Thr202/Tyr204 and a more sustained phosphorylation of MEK1/2 at residues Ser217/221 (Supplementary Fig. S1g,h). Importantly FBS-induced phosphorylation of both MEK1/2 and ERK1/2 was reduced in cells lacking PI3K-C2β (Supplementary Fig. S1g,h). Validation of these results in distinct PI3K-C2β knockdown clones indicated a significant reduction of MEK1/2 phosphorylation upon 5 and 10 min of FBS stimulation (Fig. 1c,d), as confirmed by densitometry analysis (Fig. 1e,f). Similarly a clear inhibition of FBS-induced ERK1/2 phosphorylation was detected in cells lacking PI3K-C2β (Fig. 1g–j). No major differences were observed in MEK1/2 and ERK/12 phosphorylation between parental and control sh scrambled cells (Fig. 1c–j).

**Figure 2:**
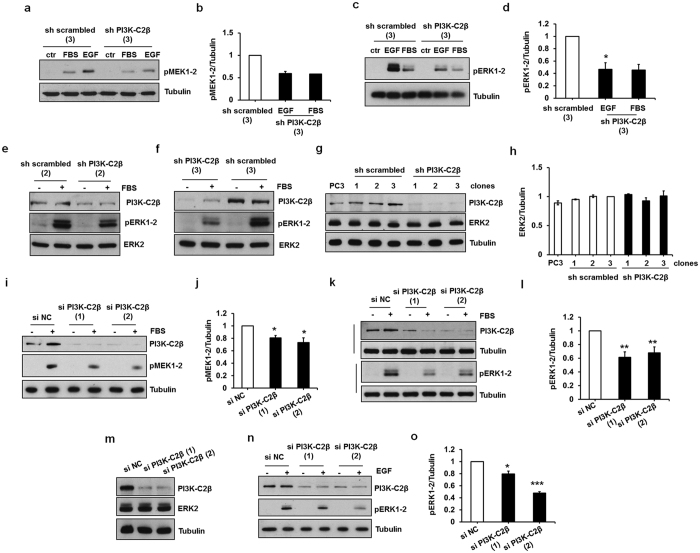
PI3K-C2β regulates EGF- and FBS-dependent MAPK activation. (**a–d**) The indicated PC3 stable clones were serum starved overnight and then stimulated with 20 ng/ml EGF for 10 min. In some of these experiments, FBS was used as positive control. Phosphorylation of MEK1/2 (**a,b**) and ERK1/2 (**c,d**) was assessed by Western blotting. Results from densitometry analysis are expressed as fold change of the sh scrambled clone and are means ± s.e.m. from the following number of experiments: (**b**) n = 2 (EGF), n = 1 (FBS); (**d**): n = 3 (EGF), n = 2 (FBS). *p < 0.05. (**e,f**) The indicated PC3 stable clones were treated as above. Membranes were then stripped and re-incubated with total ERK2. (**g,h**) Total levels of ERK2 were assessed in PC3 and in the indicated stable clones. Results from densitometry analysis are expressed as fold change of sh scrambled clone 3 and are means ± s.e.m. from the following number of experiments: n = 2 (sh scrambled clone 1, sh PI3K-C2β clone 1), n = 3 (sh scrambled clone 2), n = 4 (PC3), n = 5 (sh scrambled clone 3, sh PI3K-C2β clones 2 and 3). (**i–m**) PC3 cells were transfected with two specific siRNAs targeting PI3K-C2β and a non targeting, negative control siRNA (si NC), serum starved overnight the following day and then stimulated with FBS for 10 min. Phosphorylation of MEK1/2 (**i,j**), phosphorylation of ERK1/2 (**k,l**) and total levels of ERK2 (**m**) were assessed by Western blotting. Results from densitometry analysis are expressed as fold change of cells transfected with si NC and are means ± s.e.m. from n = 3 (**j**), n = 5–6 (**l**) independent experiments. *p < 0.05, **p < 0.01. (**n,o**) LNCaP cells were transfected with the indicated siRNAs, serum starved and then stimulated with 20 ng/ml EGF for 10 min. Phosphorylation of ERK1/2 was assessed by Western blotting. Results from densitometry are means ± s.e.m. from n = 3 independent experiments. *p < 0.05, ***p < 0.001. In all panels representative blots are shown. Where indicated tubulin was used as loading control and levels of PI3K-C2β were also assessed in the same or parallel gels.

To further validate the role of PI3K-C2β in ERK1/2 activation stable PC3 clones were stimulated with epidermal growth factor (EGF). Consistent with data obtained upon FBS stimulation, EGF-induced MEK1/2 (Fig. 2a,b) and ERK1/2 (Fig. 2c,d) phosphorylation was strongly reduced in cells lacking PI3K-C2β. Importantly the reduced phosphorylation was not due to reduced total ERK levels in sh PI3K-C2β stable cell lines (Fig. 2e–h, Supplementary Fig. S2a).

Taken together these data indicate that PI3K-C2β regulates MEK/ERK activation upon FBS and EGF stimulation.

### Transient PI3K-C2β downregulation inhibits MEK1/2 and ERK1/2 activation

In order to rule out any potential non-specific effect due to the stable downregulation of PI3K-C2β we next investigated the effect of transient downregulation of the enzyme on MEK1/2 and ERK1/2 phosphorylation. Consistent with data obtained using the stable clones, PI3K-C2β downregulation using two distinct siRNAs significantly inhibited FBS-induced MEK1/2 (Fig. 2i,j) and ERK1/2 phosphorylation (Fig. 2k,l) compared to cells transfected with a non targeting, negative control siRNA (si NC). As for stable cells, transient downregulation of PI3K-C2β did not reduce total ERK levels (Fig. 2m).

To further investigate the role of PI3K-C2β in MAPK activation in PCa cells, we determined the effect of its transient downregulation in LNCaP cells that express high levels of PI3K-C2β and do not express PTEN (Fig. 1a). Preliminary experiments revealed a strong phosphorylation of ERK1/2 upon EGF stimulation in these cells (Supplementary Fig. S2b). Consistent with data in PC3, the EGF-induced ERK1/2 phosphorylation was significantly reduced in LNCaP upon transient downregulation of PI3K-C2β with two distinct siRNAs (Fig. 2n,o).

We next investigated whether other PI3K isoforms were also involved in regulation of ERK phosphorylation. To this end PC3 cells were treated with the PI3K inhibitor LY294002 before stimulation with FBS or EGF. The concentration of LY294002 used (5 μM) is reported not to affect PI3K-C2β activation^31^,^39^ while being able to inhibit class I and class III PI3K isoforms. Indeed treatment of cells with LY294002 (5 μM) clearly inhibited phosphorylation of Akt at its residue Ser473 (Supplementary Fig. S2c–e). In contrast the inhibitor did not affect either the FBS- or the EGF-induced ERK1/2 phosphorylation (Supplementary Fig. S2f–h) suggesting that class I and class III PI3Ks are not involved in ERK1/2 activation in PC3 cells.

Taken together these data indicate a specific role for PI3K-C2β in regulation of MEK/ERK activation in PCa cells.

### The PI3K-C2β/ERK pathway does not regulate cell growth in PCa cells

**Figure 3:**
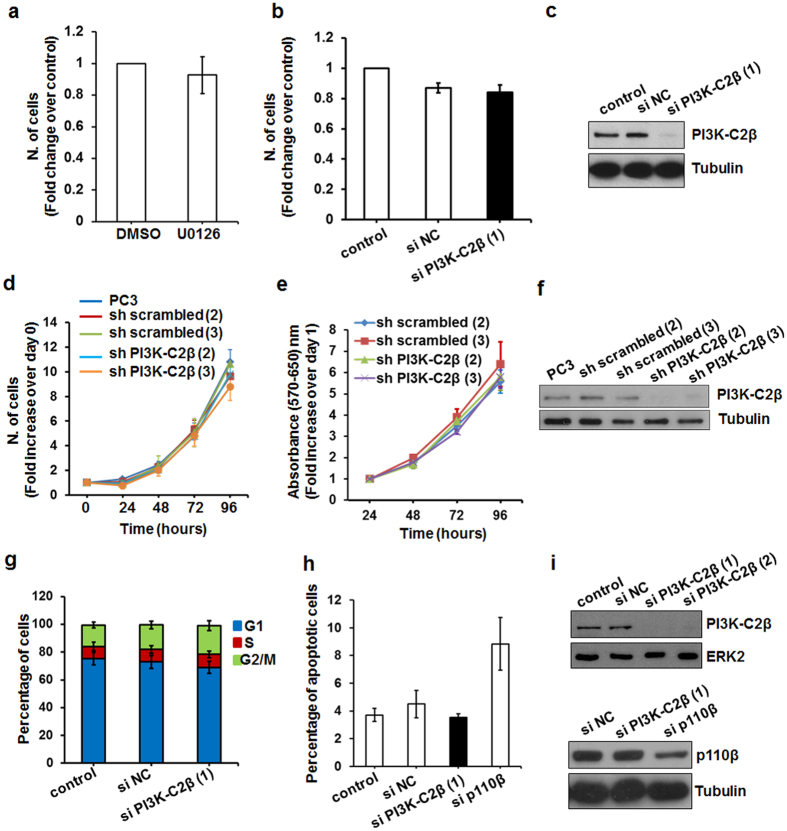
MEK/ERK and PI3K-C2β are not required for PCa cell proliferation. (**a**) PC3 cells were incubated in growth media supplemented with MEK1/2 inhibitor U0126 (25 μM) or vehicle control (DMSO) for 72 h before counting. Data are expressed as fold change of number of cells treated with DMSO and are means ± s.e.m. from n = 3 independent experiments. (**b,c**) PC3 cells were transiently transfected with a specific siRNA targeting PI3K-C2β (sequence 1), a non targeting, negative control siRNA (si NC) or transfection reagent (control) alone. The number of cells was determined by manual counting after 72 h in growth media. Data are expressed as fold change of number of control cells and are means ± s.e.m. of n = 4 independent experiments. Efficient downregulation of PI3K-C2β was confirmed by Western blotting analysis. Tubulin was used as loading control. (**d**) Data from cell counting are expressed as fold change of number of cells plated at day 0 and are means ± s.e.m. of n = 7 (PC3), n = 11 (sh scrambled clone 2), n = 3–4 (sh scrambled clone 3), n = 8 (sh PI3K-C2β clone 2) and n = 5–6 (sh PI3K-C2β clone 3) independent experiments. (**e**) Data from MTT assay are expressed as fold change of absorbance determined at day 1 and are means ± s.e.m. of n = 3–4 experiments. (**f**) Representative blot showing expression levels of PI3K-C2β in the indicated cell lines. (**g**) PC3 cells were transiently transfected as before and fixed after 72 h. Cell cycle analysis was performed by FACS as described in the Methods section. Data are expressed as percentage of cells in the distinct phases and are means ± s.e.m. of n = 4 independent experiments. (**h**) PC3 cells were transiently transfected with the indicated siRNAs. The percentage of apoptotic cells was determined 48 h after transfection by Annexin V/FACS analysis as described in the Methods section. Data are expressed as percentage of early + late apoptotic cells and are means ± s.e.m. of n = 3–4 independent experiments. Data from one experiment with siRNA PI3K-C2β (sequence 1) and two experiments with siRNA PI3K-C2β (sequence 2) are pulled together. (**i**) Efficiency of PI3K-C2β and p110β downregulation was assessed by Western blotting.

We next investigated whether the PI3K-C2β-dependent MEK/ERK activation had a role on PCa cell growth. Interestingly, incubation of PC3 cells for 72 h in growth media supplemented with the MEK1/2 inhibitor U0126 did not reduce the number of cells (Fig. 3a). Similarly transient downregulation of PI3K-C2β did not reduce the number of cells as determined 72 h following transfection (Fig. 3b). Efficiency of PI3K-C2β downregulation was assessed by Western blotting analysis (Fig. 3c). Consistent with this, cell counting (Fig. 3d) and MTT assays (Fig. 3e) revealed no difference in proliferation in growth media between parental PC3, two distinct sh scrambled and two distinct sh PI3K-C2β stable clones. Levels of PI3K-C2β in the indicated clones was assessed by Western blotting analysis (Fig. 3f). Furthermore no statistically significant difference in the percentage of cells in the G1, S and G2/M phases of the cell cycle (Fig. 3g) or in the percentage of apoptotic cells (Fig. 3h) was detected in PC3 cells upon transient downregulation of PI3K-C2β. As a control downregulation of the class I PI3K isoform p110β performed in parallel was clearly able to induce apoptosis in these cells (Fig. 3h). Western blotting analysis confirmed efficient downregulation of PI3K-C2β and p110β (Fig. 3i).

Taken together these data indicate that the PI3K-C2β/MEK/ERK pathway does not regulate PC3 cell proliferation, at least in normal growing conditions.

### MEK/ERK and PI3K-C2β are involved in PCa invasion

**Figure 4:**
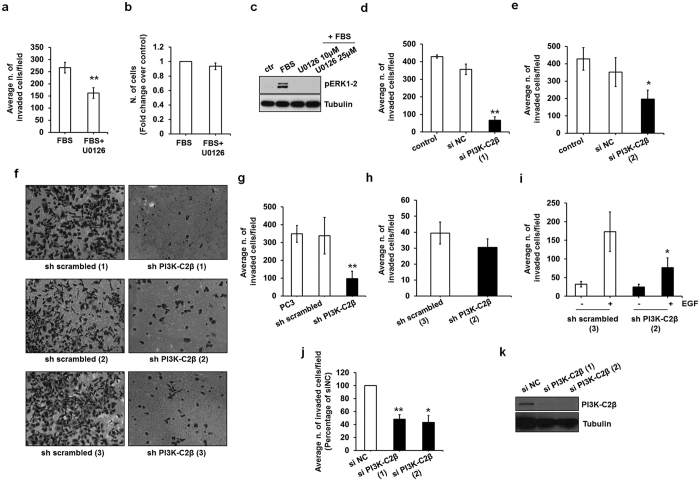
PI3K-C2β is involved in PCa cell invasion. (**a,b**) Serum starved PC3 cells were pre-treated with U0126 (25 μM) or vehicle alone (DMSO) for 30 min, detached and plated for FBS-induced invasion. Data in (**a**) are means ± s.e.m. from n = 3 independent experiments performed in duplicate. **p < 0.01. The same number of cells was plated in parallel in DMEM containing 10% FBS supplemented with U0126 (25 μM) or DMSO and counted after 48 h. Data in (**b**) are expressed as fold change of number of cells treated with DMSO and are means ± s.e.m. from n = 3 independent experiments performed in duplicate. (**c**) PC3 cells were plated in parallel with cells to be used in invasion assays, starved, pre-treated with the indicated concentrations of U0126 for 30 min and then stimulated with FBS for 10 min in the presence or absence of the inhibitor. Phosphorylation of ERK1/2 was assessed by Western blotting. Tubulin was used as loading control. (**d,e**) PC3 cells were transiently transfected with the indicated siRNAs or transfection reagent alone (control), serum starved overnight the following day and then detached and plated for FBS-induced invasion assay. Data are expressed as average number of invaded cells/field and are means ± s.e.m. of n = 3 independent experiments performed in duplicate. **p < 0.01, *p < 0.05. (**f,g**) Data from FBS-induced invasion assays in all control (sh scrambled) and all knockdown (sh PI3K-C2β) clones are pulled together and compared to parental PC3 cells and are means ± s.e.m. from n = 6 (PC3) and n = 8 (stable clones) independent experiments performed in duplicate. **p < 0.01 vs sh scrambled. Representative images for each clone are also shown in (**f**). (**h**) Results from basal invasion on Matrigel are means ± s.e.m. from n = 7 independent experiments performed in duplicate. (**i**) Results from EGF-induced invasion assays in the indicated cells are means ± s.e.m. from n = 3 independent experiments performed in duplicate. *p < 0.05. (**j,k**) FBS-induced invasion in LNCaP cells transiently transfected with the indicated siRNAs. Results are expressed as percentage of invaded cells transfected with siNC and are means ± s.e.m. of n = 3 independent experiments performed in duplicate. *p < 0.05, **p < 0.01. Efficiency of PI3K-C2β downregulation was assessed by Western blotting.

Although the MEK/ERK pathway is usually associated with cell proliferation data in literature also indicate a role for these kinases in regulation of cell motility and migration. We therefore decided to determine whether this pathway was required for PC3 cell invasion. Inhibition of MEK1/2 with the specific inhibitor U0126 significantly reduced FBS-induced cell invasion (Fig. 4a) without affecting cell proliferation assessed in parallel (Fig. 4b). Efficiency of the inhibitor was confirmed by Western blotting analysis of lysates from cells plated in parallel and stimulated with FBS (Fig. 4c). These data indicate a role for MEK/ERK in PCa cell invasion.

We then investigated whether PI3K-C2β was also involved in this process. As shown in Fig. 4d,e, transient downregulation of PI3K-C2β with two distinct siRNAs strongly reduced FBS-induced cell invasion in PC3 cells. No statistically significant difference was observed in invasion of cells transfected with control siRNA (si NC) compared to cells (control) treated with the transfection reagent alone (Fig. 4d,e). A strong inhibition of FBS-induced invasion was also detected in three distinct sh PI3K-C2β stable clones compared to control sh scrambled cells (Fig. 4f,g). Representative images are shown in Fig. 4f. Quantification of these assays indicated a very strong inhibition of invasion in cells lacking PI3K-C2β (Fig. 4g). No difference was detected between stable control cells compared to parental PC3 cells (Fig. 4g). On the other hand downregulation of PI3K-C2β did not affect basal invasion induced by Matrigel alone (Fig. 4h), indicating a specific role for PI3K-C2β in growth factors-induced cell invasion. Consistent with this, we observed that EGF-induced invasion was also reduced in cells lacking PI3K-C2β (Fig. 4i). In addition downregulation of PI3K-C2β using two distinct siRNAs reduced the FBS-induced invasion in LNCaP cells (Fig. 4j). Efficient downregulation of PI3K-C2β in this cell line was confirmed by Western blotting (Fig. 4k).

Taken together these data indicate that MEK/ERK and PI3K-C2β are required for PCa cell invasion.

### PI3K-C2β regulates PCa cell migration

**Figure 5:**
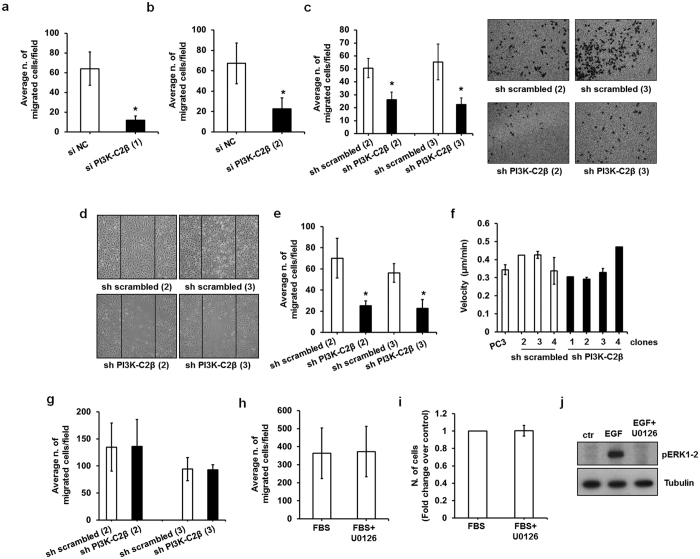
PI3K-C2β is involved in PCa cell migration. (**a,b**) PC3 cells were transiently transfected with the indicated siRNAs. FBS-induced cell migration was assessed by Transwell assays. Data are means ± s.e.m. of n = 3 independent experiments performed in duplicate. *p < 0.05. (**c**) Results from FBS-induced migration assays in the indicated stable PC3 clones. Data are means ± s.e.m. of n = 3 independent experiments performed in duplicate. *p < 0.05. Representative images of Transwell inserts are also shown. (**d,e**) Representative images (**d**) and results (**e**) from wound healing assays in the indicated stable PC3 clones. Data are expressed as number of cells migrated into the wounded area and are means ± s.e.m. of n = 4 independent experiments. *p < 0.05. (**f**) Results from random motility assays in the indicated stable PC3 clones. Data indicate the mean velocity of single cells and are means ± s.e.m. of n = 3 (PC3, sh scrambled clones 3,4 and sh PI3K-C2β clone 3), n = 2 (sh PI3K-C2β clone 2) and n = 1 (sh scrambled clone 2, sh PI3K-C2β clones 2,4) independent experiments. (**g**) Results from Type I collagen-induced migration assays in the indicated stable PC3 clones. Data are means ± s.e.m. of n = 3 independent experiments performed in duplicate. (**h**) Serum starved PC3 cells were pre-treated with U0126 (25 μM) or vehicle alone for 30 min before being detached and plated for FBS-induced migration assays in the absence or presence of the inhibitor. Data are means ± s.e.m. of n = 3 independent experiments performed in duplicate. (**i**) PC3 detached for cell migration as in (**h**) were re-plated in DMEM containing 10% FBS supplemented with U0126 or vehicle alone. Cells were counted at the end of the migration assays. Data are means ± s.e.m. of n = 3 independent experiments performed in duplicate. (**j**) PC3 cells were plated in parallel with cells to be used in migration assays, starved, pre-treated with the indicated concentrations of U0126 for 30 min and then stimulated with EGF (20 ng/ml) in the presence or absence of the inhibitor for 10 min. Activation of ERK1/2 was assessed by Western blot. Tubulin was used as loading control.

Several studies have reported that PI3K-C2β regulates cell migration in different cell types^31^,^32^,^33^,^37^,^40^. Therefore we next investigated whether the effect of PI3K-C2β downregulation on PCa cell invasion was due to a specific role of the enzyme in regulation of cell migration in these cells. Transwell assays revealed a clear reduction in FBS-induced migration of PC3 cells upon downregulation of PI3K-C2β using two distinct siRNAs (Fig. 5a,b). Similarly, reduced migration was observed in two distinct sh PI3K-C2β clones compared to sh scrambled cell lines (Fig. 5c). Representative images of migrated cells are also shown in Fig. 5c. Consistent with data from Transwell assays, cells lacking PI3K-C2β also displayed impaired FBS-induced migration in wound healing assays (Fig. 5d,e). On the other hand no major differences were detected in the distance and velocity of parental and stable cell lines in single cell-motility assays (Fig. 5f), consistent with previous observations in endothelial cells^40^. Similarly downregulation of PI3K-C2β did not affect migration induced by Type I collagen (Fig. 5g), indicating a specific role for this enzyme in FBS-dependent PCa cell migration.

We then determined whether MEK/ERK were also involved in this process. Interestingly treatment of cells with the MEK1/2 inhibitor U0126 did not affect the FBS-induced migration of PC3 cells assessed by Transwell assays (Fig. 5h). Consistent with data presented above (Figs 3a and 4b) incubation with the inhibitor did not affect cell numbers (Fig. 5i). Efficiency of the inhibitor was confirmed in experiments performed in parallel (Fig. 5j). These data demonstrate that MEK/ERK are involved in invasion but not migration of PCa cells and suggest that PI3K-C2β can control cell invasion partly by regulating the MEK/ERK pathway and partly by activating additional signalling pathways that modulate PCa cell migration.

### PI3K-C2β specifically regulates the expression levels of the transcription factor Slug

**Figure 6:**
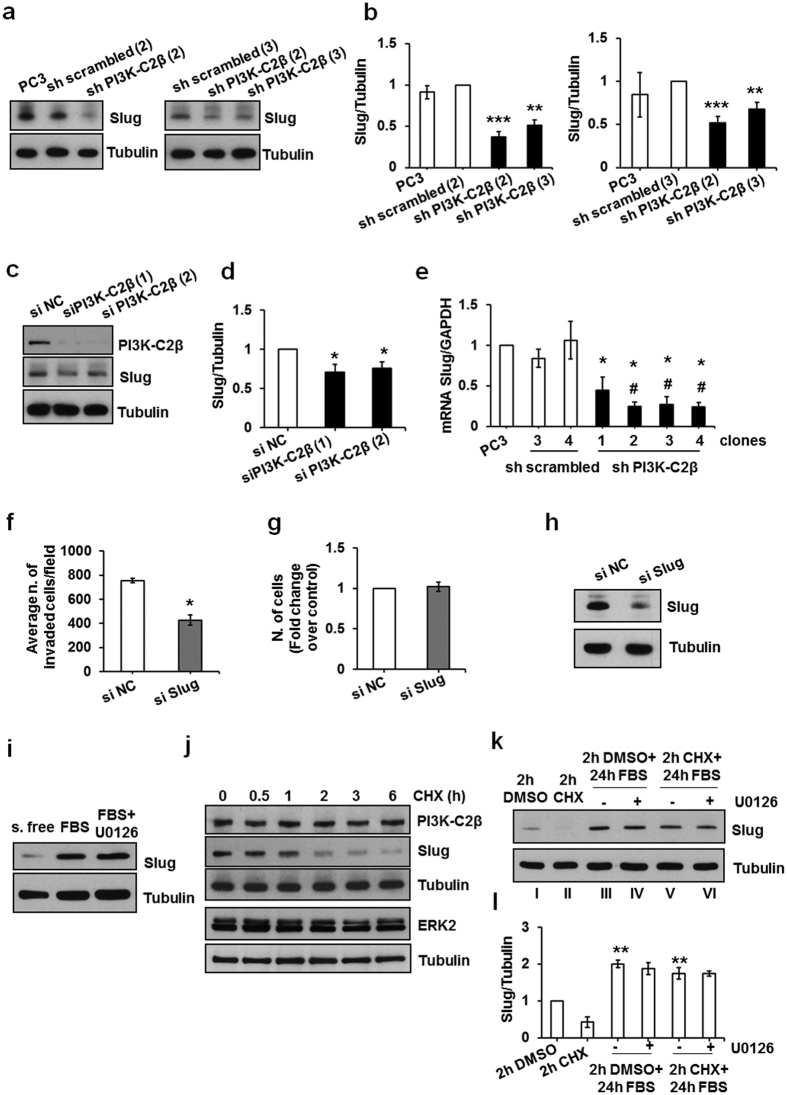
PI3K-Cβ regulates levels of the transcription factor Slug. (**a,b**) Representative blots and results from densitometry analysis expressed as fold change of normalised levels of Slug in the shscrambled clone used in each set of experiments. Data are means ± s.e.m. from n = 5–10 independent experiments. **p < 0.01, ***p < 0.001 vs shscrambled. (**c,d**) Representative blots and results from densitometry analysis expressed as fold change of normalised levels of Slug in cells transfected with si NC. Data are means ± s.e.m. from: n = 4–5 independent experiments. *p < 0.05. (**e**) Results from qPCR analysis expressed as fold change of normalised levels of Slug mRNA in PC3. Data are means ± s.e.m. from n = 3 independent experiments. shPI3K-C2β clone 1: *p < 0.05 vs PC3 and shscrambled clone 4; shPI3K-C2β clone 2: *p < 0.05 vs shscrambled clone 4, ^#^p < 0.01 vs PC3 and shscrambled clone 2; shPI3K-C2β clone 3: *p < 0.05 vs shscrambled clone 4 and shscrambled clone 2, ^#^p < 0.01 vs PC3; shPI3K-C2β clone 4: *p < 0.05 vs shscrambled clone 4, ^#^p < 0.01 vs PC3 and shscrambled clone 2. (**f**) Data from FBS-induced invasion assays in transiently transfected PC3 cells are expressed as average number of invaded cells/field and are means ± s.e.m. of n = 3 independent experiments performed in duplicate. *p < 0.05. (**g**) Data from cell counting performed in parallel with invasion assays are means ± s.e.m. of n = 4 independent experiments in duplicate. (**h**) Efficient downregulation of Slug was assessed by Western blotting. (**i**) Serum starved PC3 cells were lysed (s.free) or incubated in FBS for 24 h in the presence or absence of U0126 (25 μM) before lysis. (**j**) Serum starved PC3 were incubated with 50 μg/ml cycloheximide (CHX) or vehicle (DMSO) for the indicated times before lysis. (**k,l**) Serum starved PC3 cells were incubated with 50 μg/ml CHX or DMSO for 2 h and either lysed or incubated in FBS in the presence or absence of U0126 (25 μM) for further 24 h before lysis. Data from densitometry analysis are expressed as fold change of normalised levels of Slug in cells treated with DMSO for 2 h and are means ± s.e.m. from n = 3 independent experiments. In (**j,k**) tubulin was used as loading control.

In an effort to further characterise the signalling pathways regulated by PI3K-C2β and involved in cell migration/invasion we next determined whether downregulation of this enzyme might affect the expression levels of proteins involved in epithelial-mesenchymal transition (EMT), a process by which epithelial cells can acquire migratory and invasive properties^41^. No major differences in the expression levels of the transcription factors TCF8/ZEB1 (Supplementary Fig. S3a,b) and ZO-1 (Supplementary Fig. S3d,e), β catenin (Supplementary Fig. S3a,c), Vimentin (Supplementary Fig. S3d,f) and Claudin-1 (Supplementary Fig. S3g,h) was detected in sh PI3K-C2β compared to sh scrambled cells by Western blotting analysis. Similarly downregulation of PI3K-C2β did not affect mRNA levels of β catenin (Supplementary Fig. S3i), Vimentin (Supplementary Fig. S3j) and Twist (Supplementary Fig. S3k) as assessed by qPCR analysis. On the other hand Western blotting analysis revealed a slight but significant reduction in the levels of the transcription factor Slug in stable sh PI3K-C2β clones compared to parental PC3 and sh scrambled cells (Fig. 6a,b). These data were confirmed upon transient downregulation of PI3K-C2β (Fig. 6c,d). Reduced levels of Slug mRNA were detected in all four sh PI3K-C2β stable clones (Fig. 6e). To determine whether reduced levels of Slug were associated with impaired cell invasion PC3 cells were transfected with a siRNA specifically targeting the transcription factor (Fig. 6f–h). Consistent with previous reports^42^,^43^ downregulation of Slug significantly inhibited cell invasion (Fig. 6f) without affecting the total number of cells determined in parallel (Fig. 6g). Western blotting analysis confirmed reduced Slug levels in cells upon transfection (Fig. 6h). These data suggest that PI3K-C2β might further impact on cell migration and ultimately cell invasion by regulating Slug expression.

### The PI3K-C2β-dependent regulation of Slug does not involve MEK/ERK activation

It has been recently reported that lipocalin 2 can induce Slug expression in 22RV1 PCa cells in a mechanism involving ERK activation^44^. We therefore determined whether MEK/ERK were also involved in Slug regulation in PC3 cells and whether the reduced Slug levels detected in stable PI3K-C2β knockdown clones was associated with the reduced FBS-dependent ERK activation observed in the same cells. First we observed that FBS stimulation for 24 h following overnight starvation was able to increase Slug expression levels in PC3 cells (Fig. 6i). In these conditions addition of U0126 did not appear to prevent stimulation of Slug expression (Fig. 6i). To better investigate modulation of Slug expression levels, serum-starved PC3 cells were incubated with cycloheximide (CHX) to inhibit protein synthesis for different times (Fig. 6j). We observed that incubation with CHX for 2 h was already able to strongly reduce Slug levels without affecting PI3K-C2β or ERK2 levels (Fig. 6j). We then treated serum starved PC3 cells for 2 h with CHX or vehicle (DMSO) alone before incubating them for 24 h in FBS in the presence or absence of MEK inhibitor U0126. Stimulation of cells with FBS for 24 h following 2 h treatment with DMSO or CHX strongly increased Slug protein levels (Fig. 6k,l - lanes I/III, II/V). In both cases simultaneous incubation with U0126 did not prevent the FBS-induced stimulation of Slug expression (Fig. 6k,l - lanes IV and VI), ruling out a main role for MEK/ERK in regulation of Slug expression in PC3 cells. Taken together these data suggest that PI3K-C2β modulates PCa cell migration and invasion by regulating distinct cellular processes, including MEK/ERK activation and expression levels of the transcription factor Slug.

## Discussion

Recent studies have greatly improved our understanding of the physiological roles of class II PI3K isoforms^17^ and they have contributed to establish that these enzymes are not redundant and can regulate distinct cellular functions compared to other PI3Ks^2^. Despite this there is still a limited understanding of the signalling pathways that can be specifically regulated by each class II PI3K isoform.

Here we demonstrate that PI3K-C2β regulates FBS- and EGF-induced MEK/ERK activation in PCa cell lines. The observation that activation of ERK1/2 was not affected by treatment with LY294002 at a concentration able to inhibit class I and class III PI3Ks but not PI3K-C2β suggest a specific role for this isoform in MEK/ERK regulation. There are contrasting data in literature on the role of PI3K-C2β in ERK1/2 regulation. It was originally reported that transfection of HEK293 or COS-7 cells with either wild-type PI3KC2β or a kinase inactive mutant PI3KC2βDN construct did not affect EGF-induced ERK activation^35^. Similarly overexpression of PI3KC2βDN in H-69 SCLC cells did not inhibit the stem cell factor-induced ERK1/2 phosphorylation or activation assessed by *in vitro* kinase assay^35^. Consistent with these data, no effect on sphingosine 1-phosphate-dependent ERK1/2 phosphorylation was detected in human umbilical vein endothelial cells upon downregulation of PI3KC2β using transient transfection of specific siRNA^40^ or in EGF-mediated ERK1/2 phosphorylation in SK-N-AS and IMR-5 neuroblastoma cell lines stably infected with shRNAs targeting PI3KC2β^34^. On the other hand both basal and EGF-mediated ERK1/2 activation appeared to be inhibited in A-431 cells overexpressing either wild type or kinase dead PI3KC2β D1213A-17 and D1213A-32^32^. Furthermore EGF- or platelet derived growth factor-induced ERK1/2 phosphorylation was increased in NIH3T3 overexpressing PI3KC2β and reduced in NIH3T3 overexpressing PI3KC2βDN^36^. Our data here indicate a specific role for PI3K-C2β in regulation of MEK/ERK in PCa cell lines PC3 and LNCaP. It is worth mentioning that PC3 and LNCaP are both PTEN null cells and appear to express increased levels of PI3K-C2β compared to the PTEN-expressing PCa cell line DU145 or the prostate cell line PNT2. Whether PI3K-C2β specifically regulates MEK/ERK in the context of PTEN deletion/mutation remains to be established.

We further show that downregulation of PI3K-C2β inhibited PCa cell invasion. While data have previously indicated a role for this enzyme in migration of several cell types^31^,^32^,^33^,^37^,^40^ this is the first study demonstrating that PI3K-C2β is required for PCa cell invasion. Importantly we observed that inhibition of MEK/ERK also reduced PCa cell invasion, consistent with a previous study suggesting that downregulation of ERK2 in PC3-ML, a subclone of PC3 able to metastasise to the lumbar vertebrae, resulted in reduced ability to form metastasis^45^. Taken together these data indicate that the PI3K-C2β-dependent MEK/ERK regulation is involved in regulation of PCa cell invasion. On the other hand neither PI3K-C2β nor MEK/ERK appear to play a main role in PCa cell proliferation, at least in normal growing conditions. Interestingly we observed that downregulation of PI3K-C2β resulted in a much stronger reduction of cell invasion compared to the effect of MEK/ERK inhibition. Indeed further investigation revealed that downregulation of PI3K-C2β reduced both FBS-induced cell migration and invasion whereas MEK/ERK inhibition impaired cell invasion without affecting cell migration. This is consistent with a previous report demonstrating that ERK1/2 is not involved in EGF-induced migration of prostate cancer cell line DU145^46^ and suggests that the PI3K-C2β-regulation of MEK/ERK is involved in steps necessary for cell invasion other than cell migration. In this respect it is noteworthy that ERK1/2 has been previously reported to play a key role in ADAM17-dependent invasion of PCa cell through overexpression of metalloproteinases MMP-2 and MMP-9^47^.

Taken together these data suggest that PI3K-C2β can control cell invasion partly by regulating the MEK/ERK pathway and partly by activating additional signalling pathways that modulate PCa cell migration. It has been previously reported that PI3K-C2β can regulate cell motility by modulating activation of GTPases and actin remodelling^32^. Indeed PI3K-C2β has been recently identified as the main PI3K isoform involved in actin remodelling of ovarian cancer cells^33^ confirming its critical role in regulation of migration of these cells^31^,^33^. To further define the mechanisms involved in PI3K-C2β-dependent regulation of cell migration we investigated the potential contribution of this enzyme to EMT and we observed that downregulation of PI3K-C2β induced a specific reduction in the expression levels of the transcription factor Slug. Slug is a member of the Snail super family of zinc finger transcription factors that have a key role in EMT and in regulation of cell invasion and metastasis in several cancer types^48^,^49^,^50^. In particular we observed that downregulation of Slug inhibited invasion of PC3 cells, consistent with a previous study^42^ and with a report demonstrating that overexpression of Slug stimulates migration and invasion of PC3 cells^43^. Moreover it was proposed that inhibition of Slug might represent one of the main mechanisms by which combination of mTOR, ERK1/2 and Hsp90 inhibitors was able to reduce circulating tumour cells in xenograft mouse models implanted with PCa cell lines^51^. Here we show that downregulation of PI3K-C2β results in reduced expression levels of Slug in PC3 cells and this is the first evidence of a role for a class II PI3K in regulation of the expression of this transcription factor. These results together with our and previous data demonstrating the role of Slug in migration and invasion strongly suggest that modulation of Slug levels may be one of the mechanisms by which PI3K-C2β regulates these cellular processes. Furthermore, as we ruled out a main role for MEK/ERK in Slug regulation in PC3 cells, these data demonstrate that PI3K-C2β can control PCa cell migration and invasion through different cellular pathways.

Taken together our study has identified novel signalling pathways specifically activated by PI3K-C2β and it has revealed an important role for this enzyme in PCa cells. It has been estimated that roughly 40% of primary and 70% of metastatic PCa have genomic alterations of PI3K-dependent signalling pathway^52^. Complete loss or haploinsufficiency of PTEN occurs in 20–70% of primary PCa and 30–80% of locally advanced and metastatic disease^53^. Animal models have indicated the pivotal role of PTEN in PCa^54^,^55^,^56^. Homozygous *Pten*-null mice develop prostate intraepithelial neoplasia (PIN) that do not evolve to more aggressive forms of the disease mostly because of simultaneous development of other tumours. On the other hand, homozygous deletion of *Pten* in the mouse prostate led to PIN lesions at 6 weeks of age that progressed to invasive and metastatic prostate carcinoma within a few weeks^55^. The use of *Pten* hypomorphic alleles demonstrated that decreasing PTEN levels correlates with increased progression of prostate tumours in mice. Furthermore mouse models have also established that concurrent *Pten* hemizygosity coupled with deletions in other genes accelerates tumourigenesis. Further animal models revealed the critical role of the class I PI3K isoform p110β and not p110α in PTEN deletion-driven PCa development^57^. Indeed overexpression of p110β was able to induce early lesions of prostatic tumour formation in mice^58^. Several *in vitro* studies further confirmed the role of this enzyme and indicated a role in androgen-dependent gene expression, cell proliferation and survival^59^. Indeed a specific p110β inhibitor that has been modified to selectively target PCa cells has recently shown a very strong activity towards xenografts from different PCa cells^60^. As a consequence p110β is considered a very promising novel target in PCa^59^. Interestingly a recent study investigating the associations between single nucleotide polymorphisms in PI3K genes and PCa risk reported significant associations between a cluster of variants located upstream and in the promoter regions of *PIK3C2B*, the gene encoding for PI3K-C2β, and PCa risk^38^. Despite this interesting evidence, very little was known on the role of PI3K-C2β in PCa. Our data here demonstrate that PI3K-C2β is also a key enzyme in PCa, playing distinct roles and activating distinct cellular pathways compared to class I isoforms. Specifically, our data indicate that, while p110β has a key role in PCa cell proliferation and survival, PI3K-C2β is specifically involved in regulation of cell migration and invasion. These data suggest that PI3K-C2β may represent a novel potential target in PCa possibly to counteract metastasis progression. It is noteworthy that inhibition of this enzyme has been recently proved able to reduce ovarian cancer-derived metastasis formation in mice^33^, providing the first evidence of the anti-metastatic potential of targeting this enzyme.

In conclusion our study not only has identified novel signalling pathways specifically regulated by the class II PI3K isoform PI3K-C2β but it has also revealed a key role for this enzyme in PCa, in particular PCa cell migration and invasion.

## Methods

### Materials

Western blotting analyses were performed using the following antibodies: anti PI3K-C2α, anti PI3K-C2β (BD Transduction Laboratories); anti p110β, anti p110α, anti pERK1-2 (Thr202-Tyr204), anti pMEK1-2 (Ser217-221), anti PTEN, anti TCF8/ZEB1, anti ZO-1, anti β catenin, anti Vimentin, anti Claudin-1, anti Slug (Cell Signaling Technology); anti Tubulin, anti ERK2, anti Actin (Santa Cruz Biotechnology); anti GAPDH (Abcam); anti-rabbit IgG, anti-mouse IgG (Sigma Aldrich, UK). Transient downregulation of enzymes of interest was obtained using the following siRNAs: PI3K-C2β (sequence 1): AAGAATGCGACGCCTGGCAAG (Qiagen); PI3K-C2β (sequence 2): Cat. No. J-006772-08 (Dharmacon); p110β (Dharmacon, smartpoolA); Slug: Cat. No. J-017386-05 (Dharmacon). Non-targeting siRNAs (Ambion or Dharmacon), designated as ‘si NC’, were used as control.

### Cell lines and transfections

PC3 and LNCaP cell lines were maintained in RPMI-1640 or Dulbecco’s modified Eagle’s medium (DMEM), DU145 cells in DMEM and PNT2 cells in RPMI-1640 without phenol red. Growth medium was supplemented with 10% (v/v) FBS, 1% (v/v) of 1X penicillin/streptomycin and glutamine and 1% (v/v) sodium pyruvate. All reagents were from Life Technologies. Cells were seeded in cell culture plates and left to grow in a humidified incubator at 37 °C, 5% CO_2_ atmosphere. Transient transfections of siRNAs were performed using OligofectAMINE (Life Technologies) according to the manufacturer’s instruction. To generate stable PC3 clones, cells were transfected with pSuper-based recombinant vectors containing either a shRNA specifically targeting PI3K-C2β (based on siRNA sequence 1) or a non-targeting shRNA (designated as ‘shRNA scrambled’) using LipofectAMINE (Life Technologies). Cell populations expressing the PI3K-C2β-targeting shRNA or shRNA scrambled were selected by incubation in growth media supplemented with 1.5 μg/ml puromycin and then allowed to grow as single clones. Individual clones were then selected, amplified and subsequently cultured in the absence of puromycin.

### Western blotting analysis

Cells were left in growth media or incubated overnight in serum free DMEM, supplemented with penicillin/streptomycin and L-glutamine before stimulation with FBS or 20 ng/ml EGF. Cells were then washed and lysed either in 2% SDS or in ice-cold lysis buffer [50 mM Tris, pH 7.5, 150 mM NaCl, 1% Triton X-100, 2 mM EDTA, 2 mM EGTA, 1 μl/ml Protease inhibitor cocktail (Sigma Aldrich), 1 μl/ml Phosphatase inhibitor cocktail A (Sigma Aldrich) and 1 μl/ml Phosphatase inhibitor cocktail B (Sigma Aldrich)]. For CHX (Sigma Aldrich) experiments, serum starved cells were incubated with serum free DMEM containing 50 μg/ml CHX or vehicle (DMSO) for the indicated times. Cells were either lysed at different times or incubated with DMEM containing 10% FBS for further 24 h. Where indicated, MEK inhibitor U0126 (25 μM) was added to the media during the 24 h incubation period. Samples were separated by SDS-PAGE and transferred to nitrocellulose membranes. Membranes were then incubated in PBS supplemented with 0.05% (v/v) Tween 20 (PBS-T) and containing 5% skimmed milk powder for 30 min-1 h at room temperature, followed by overnight incubation with primary antibodies at +4 °C. After washing with PBS-T membranes were incubated with secondary antibodies for 1 h at room temperature, washed with PBS-T and exposed to ECL reagent. Where indicated membranes were stripped by incubation at +50 °C in SDS-based stripping buffer (62.5 mM Tris HCl pH 6.8, 0.5% SDS, 0.7% β mercapthoethanol) for 30 min. Densitometry was performed using Image J software.

### Human phosphokinase array

Analysis of the phosphorylation status of several kinases and proteins was performed using Proteome Profiler Human Phospho-Kinase Array Kit (R&D Systems) according to the manufacturer’s instructions. Briefly, serum starved PC3 cells were either left untreated or incubated with FBS for 10 min. Cells were then washed in PBS and incubated with Lysis Buffer 6 for 30 min at +4 °C. Cell debris were removed by centrifugation at 14,000 × g for 5 min and protein concentration was assessed by BCA Protein Assay. After blocking in Array Buffer 1 for 1 h at room temperature each membrane was incubated with 600 μg of cell lysate overnight at +4 °C. Membranes were then washed with 1X Wash Buffer and incubated with diluted Detection Antibody Cocktail A and B for 2 h at room temperature followed by incubation with Streptavidin-HRP for additional 30 min. Membranes were then incubated with Chemi Reagent Mix and exposed on an X-ray film for 1–10 min. Densitometry analysis was performed using Image J software.

### Cell Migration Assays

#### Random motility assay

Cells were plated in 6 well plates and transferred to an inverted contrast microscope (Zeiss Welwyn Garden City, UK) with a chamber of 37 °C, 5% CO_2_. Pictures were taken every 10 min for 15 h using a 10X phase contrast objective. Images were converted to movies and the motility of the cells was determined by the program MetaMorph^®^. All experiments were performed in duplicate.

#### Wound Healing Assay

Confluent cells were serum starved overnight. The following day, cells were wounded with a linear scratch using a sterile pipette tip in the presence of growth media supplemented with 10% FBS and 0.5 μg/ml mitomycin C. Timelapse microscopy was performed for 12 h. Quantitative analysis was performed by positioning a red line at time 0 and time 12 and the number of cells that had migrated into the wound area was counted.

#### Transwell Assay

Cells were seeded in a 6 well plate and serum starved overnight. To determine the effect of transient downregulation of PI3K-C2β, cells were transfected with the specific siRNA and serum starved overnight the day following the transfection. Where indicated polycarbonate Transwell inserts (8.0 μm diameter pores, 6.5 mm diameter, Costar^®^) were coated overnight with 100 μg/ml Type I collagen (Sigma Aldrich) in PBS at +4 °C. Coated or uncoated inserts were then incubated in 500 μl RPMI supplemented with 0.5% BSA for 1 hour at 37 °C/5% CO_2_ atmosphere. Cells were resuspended in RPMI supplemented with 0.5% BSA and plated on inserts (4,000 cells for collagen-induced migration; 12,000 or 20,000 cells for FBS-induced migration). The lower chamber was filled with 500 μl of media supplemented with 10% FBS (uncoated inserts) or serum free media (coated inserts) and cells were left to migrate for 24 h (FBS) or 4 h (collagen) at 37 °C/5% CO_2_ atmosphere. Non-migrated cells were removed from the upper chamber using a cotton bud while cells that had migrated were fixed using 4% paraformaldehyde for 30 min and stained with 0.1% crystal violet solution for 15 minutes. Pictures were taken from different fields using a light microscope at 10× magnification and cells were counted using the Image J program. A minimum of 10 fields were counted. All experiments were performed in duplicate.

### Cell Invasion Assay

Cells were seeded in a 6 well plate and serum starved overnight. To determine the effect of transient downregulation of PI3K-C2β or Slug, cells were transfected with the specific siRNA and serum starved overnight the day following the transfection. The following day, Matrigel pre-coated inserts (8.0 μm diameter pores, 6.5 mm diameter, BD) were rehydrated by adding 500 μl of serum free RPMI or DMEM both in the lower and in the upper chamber for at least 1 hour at 37 °C/5% CO_2_ atmosphere. Cells were resuspended in 500 μl serum free media and homogeneously seeded in the upper chamber (30,000 cells/insert). The lower chamber was filled with 500 μl of media containing 10% FBS or serum free media supplemented with 20 ng/ml EGF. To determine basal invasion on Matrigel serum free media was added to the lower chamber. Cells were left to invade through the Matrigel for 48 h at 37 °C/5% CO_2_ atmosphere before being fixed, stained and counted as described above.

### Cell Growth and Cell Cycle Assays

#### Cell Counting

Cells were seeded in 12 well plates and left in growth media. Cells were manually counted at the indicated times using a Burker chamber and a light microscope at 10x magnification. All experiments were performed in duplicate.

#### MTT 3-(4,5-Dimethylthiazol-2-yl)-2,5-diphenyltetrazolium bromide assay

Cells were seeded in 12 well plates. At the indicated times, cells were incubated with 0.5 mg/ml of Thiazolyl Blue Tetrazolium Bromide (Sigma Aldrich) for 2 h at 37 °C, 5% CO_2_ atmosphere. After washing once with PBS, 350 μl of DMSO was added to the cells for 15 min on a shaking rotor. The absorbance was read at 570–650nm using a spectrophotometer (HT Synergy). All experiments were performed in duplicate.

#### Cell Cycle Analysis – Vindellövs Propidium Iodide staining

Cells were seeded in a 6 well plate (150,000 cells/well) and transfected with the indicated siRNAs. After 72 h, cells were washed once with PBS, detached and collected in 1.5 ml microfuge tubes and centrifuged at 1,200 rpm for 5 min. Pelleted cells were fixed in ice-cold 70% ethanol, washed three times in PBS, centrifuged for 5 min and resuspended in 500 μl Vindellövs Propidium Iodide solution (50 μg/ml). Cells were then analysed by flow cytometry collecting 20,000 events per sample using Fluorescence activated cell sorting (FACS) Diva software.

### Apoptosis assay

Cells were plated in 6 well plates (150,000 cells/well) and transfected with the indicated siRNAs. At 48 h post transfection, cells were washed once with PBS and pelleted including the supernatant of the adherent cells. The pellet was resuspended in 400 μl 1X Annexin V Binding Buffer and 2 μl of Annexin V-FITC and incubated in the dark for 15 min at room temperature. Then, 4 μl of 4’,6-diamidino-2-phenylindole (DAPI) was added and the samples were analysed by flow cytometry collecting 20,000 events per sample using FACS Diva software. A control of unstained cells was incubated only with 1X Annexin V Binding Buffer and Annexin V-FITC or DAPI in order to set the quadrants.

### qPCR analysis

RNA was extracted from cells using GeneJET RNA Purification Kit (Thermo Scientific) according to the manufacturer’s instructions. The complementary DNA (cDNA) synthesis was performed on ice using Maxima Reverse Transcriptase (Thermo Scientific) according to the manufacturer’s instructions. RT-qPCR was performed using 2X Maxima SYBR green/Fluorescein (Fermentas) qPCR mix and the ABI 7500 RT-QPCR. GAPDH cDNA was also amplified as an internal control. Primers for EMT markers were kindly provided by Prof Nullin Divecha (University of Southampton, UK). All experiments were performed in triplicate. The relative changes in gene expression quantification were calculated using the relative ddCT analysis mode of the ABI 7500 Real-Time PCR system software.

### Statistical analysis

Data are expressed as means ± s.e.m. Differences between groups were analysed by Student’s *t* test in Excel (paired, one-tailed distribution) and p < 0.05 was considered statistically significant.

## Additional Information

**How to cite this article**: Mavrommati, I. *et al*. Novel roles for class II Phosphoinositide 3-Kinase C2β in signalling pathways involved in prostate cancer cell invasion. *Sci. Rep.* **6**, 23277; doi: 10.1038/srep23277 (2016).

## Supplementary Material

Supplementary Information
