# Supplementary material for: Novel roles for class II Phosphoinositide 3-Kinase C2β in signalling pathways involved in prostate cancer cell invasion
Source: Sci Rep. 2016 Mar 17;6:23277. doi: 10.1038/srep23277 (PMC4794650; doi:10.1038/srep23277)
Supplement: Supplementary Information [file srep23277-s1.pdf]

Novel roles for class II Phosphoinositide 3-Kinase C2β in signalling pathways involved in prostate cancer cell invasion

Ioanna Mavrommati[1,3], Ouma Cisse[1], Marco Falasca[1,2] and Tania Maffucci[1,*]

SUPPLEMENTARY INFORMATION

Figure S1. Results from phosphokinase antibody array analysis. (**a-c**) Representative blots showing expression levels of PI3K-C2β and other PI3Ks in the indicated stable PC3 cells. Tubulin or ERK2 were used as loading control. (**d-f**) Phosphokinase antibody array assay and densitometry analysis for the indicated enzymes and corresponding residues. Empty bar: sh scrambled; Black bar: sh PI3K-C2β. Blue circle: p-p38α; black circle: pERK1/2; red circle: pMEK1/2. (**g,h**) Time course analysis of MEK1/2 and ERK1/2 phosphorylation in the indicated clones.

Figure S2. FBS-induced MEK/ERK activation does not require class I PI3K. (**a**) Representative blot of pERK1/2 and total ERK2 in stable PC3 clones upon FBS stimulation. (**b**) LNCaP cells were serum starved overnight and then stimulated with 20 ng/ml EGF for the indicated times. Phosphorylation of ERK1/2 was assessed by Western blotting. Tubulin was used as loading control. (**c-h**) Serum starved PC3 cells were incubated for 30 minutes with LY294002 (5 μM) and then stimulated with EGF or FBS in the presence or absence of the inhibitor. Phosphorylation of Akt at its residue Ser473 (**c-e**) and ERK1/2 (**f-h**) was assessed by Western blotting. Tubulin was used as loading control. Results from densitometry analysis are expressed as fold change of cells stimulated in the absence of the inhibitor and are means ± s.e.m. from n=3 (**d,e**), n=5 (**g**) and n=3 (**h**) independent experiments. *$p<0.05$,**$p<0.01$. In (**f**) inhibition of EGF-induced ERK1/2 phosphorylation upon treatment with U0126 (25 μM) is also shown.

Figure S3. Effect of PI3K-Cβ downregulation on EMT markers levels. (**a-h**) Representative blots and corresponding densitometry from Western blotting analysis of the indicated proteins in sh scrambled and sh PI3K-C2β clones. Tubulin or GAPDH were used as loading control. Results from densitometry analysis are expressed as fold change of normalised levels of the indicated protein in sh scrambled clone 2. In (**b,e**) data are means ± s.e.m. from n=3 independent experiments, except for sh scrambled clone 4 (n=1). In (**c**) data are means ± s.e.m. from n=4 independent experiments, except for sh scrambled clone 4 (n=1), sh scrambled clone 2 (n=5) and

sh PI3K-C2β clone 2 (n=5). In (**f**) data are means ± s.e.m. from n=3 independent experiments, except for sh scrambled clone 2 (n=4) and sh PI3K-C2β clone 2 (n=4). In (**h**) data are means ± s.e.m. from n=2 independent experiments. (**i-k**) Results from qPCR analysis of mRNA levels of the indicated proteins. GAPDH was used for normalisation. Data are expressed as fold change of normalised mRNA levels in parental PC3 cells. Data are means ± s.e.m. from n=3 independent experiments.

Figure S1:

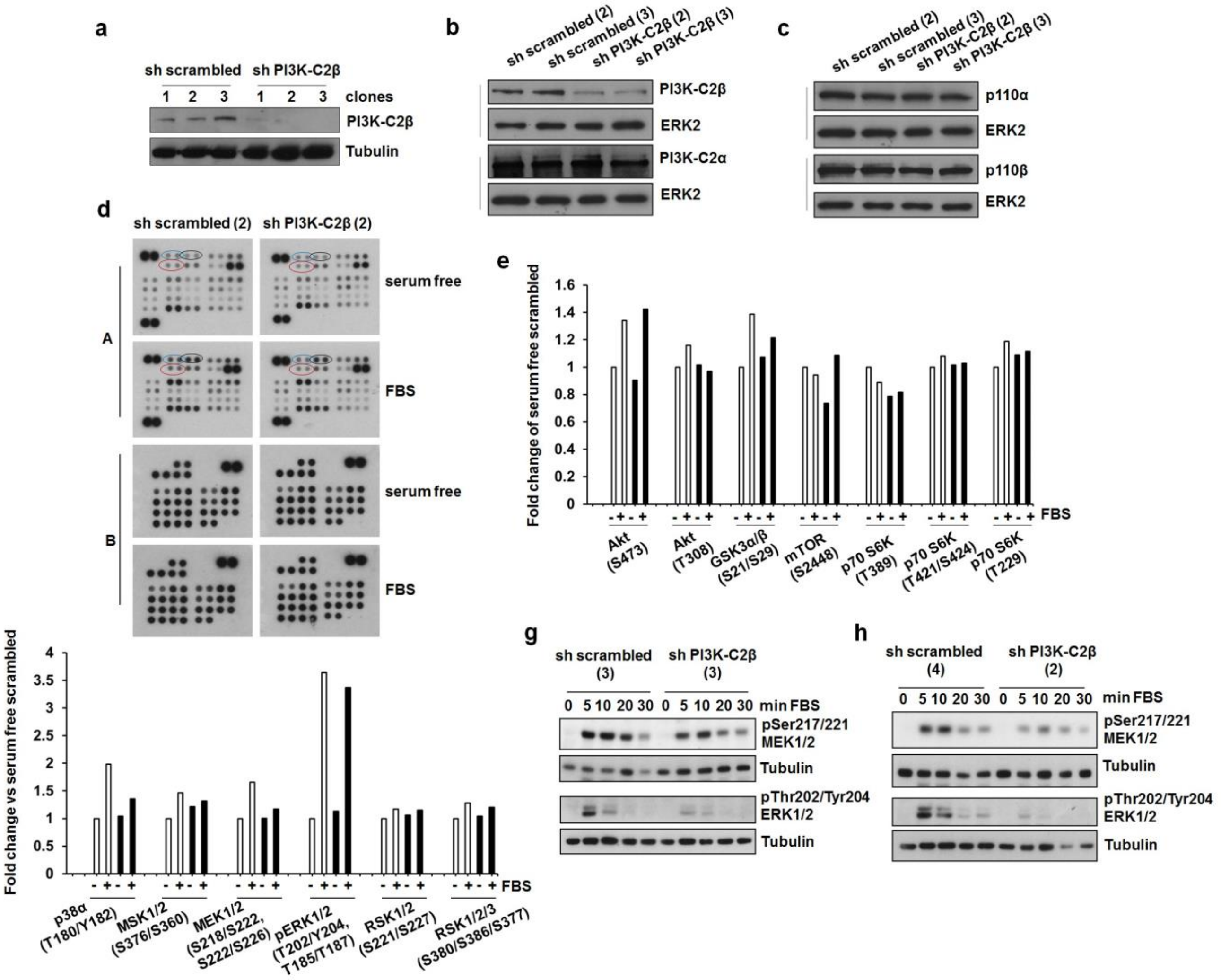
a
sh scrambled
sh PI3K-C2β
1 2 3 1 2 3 clones
PI3K-C2β
Tubulin
b
sh scrambled (2)
sh scrambled (3)
sh PI3K-C2β (2)
sh PI3K-C2β (3)
PI3K-C2β
ERK2
PI3K-C2α
ERK2
c
sh scrambled (2)
sh scrambled (3)
sh PI3K-C2β (2)
sh PI3K-C2β (3)
p110α
ERK2
p110β
ERK2
d
sh scrambled (2)
sh PI3K-C2β (2)
A
B
serum free
FBS
serum free
FBS
e
Fold change of serum free scrambled
1.6
1.4
1.2
1
0.8
0.6
0.4
0.2
0
- + - +
FBS
Akt (S473)
Akt (T308)
GSK3α/β (S21/S29)
mTOR (S2448)
p70 S6K (T389)
p70 S6K (T421/S424)
p70 S6K (T229)
f
Fold change vs serum free scrambled
4
3.5
3
2.5
2
1.5
1
0.5
0
- + - +
FBS
p38α (T180/Y182)
MSK1/2 (S376/S360)
MEK1/2 (S218/S222, S222/S226)
pERK1/2 (T202/Y204, T185/T187)
RSK1/2 (S221/S227)
RSK1/2/3 (S380/S386/S377)
g
sh scrambled (3)
sh PI3K-C2β (3)
0 5 10 20 30 0 5 10 20 30 min FBS
pSer217/221 MEK1/2
Tubulin
pThr202/Tyr204 ERK1/2
Tubulin
h
sh scrambled (4)
sh PI3K-C2β (2)
0 5 10 20 30 0 5 10 20 30 min FBS
pSer217/221 MEK1/2
Tubulin
pThr202/Tyr204 ERK1/2
Tubulin

Figure S2:

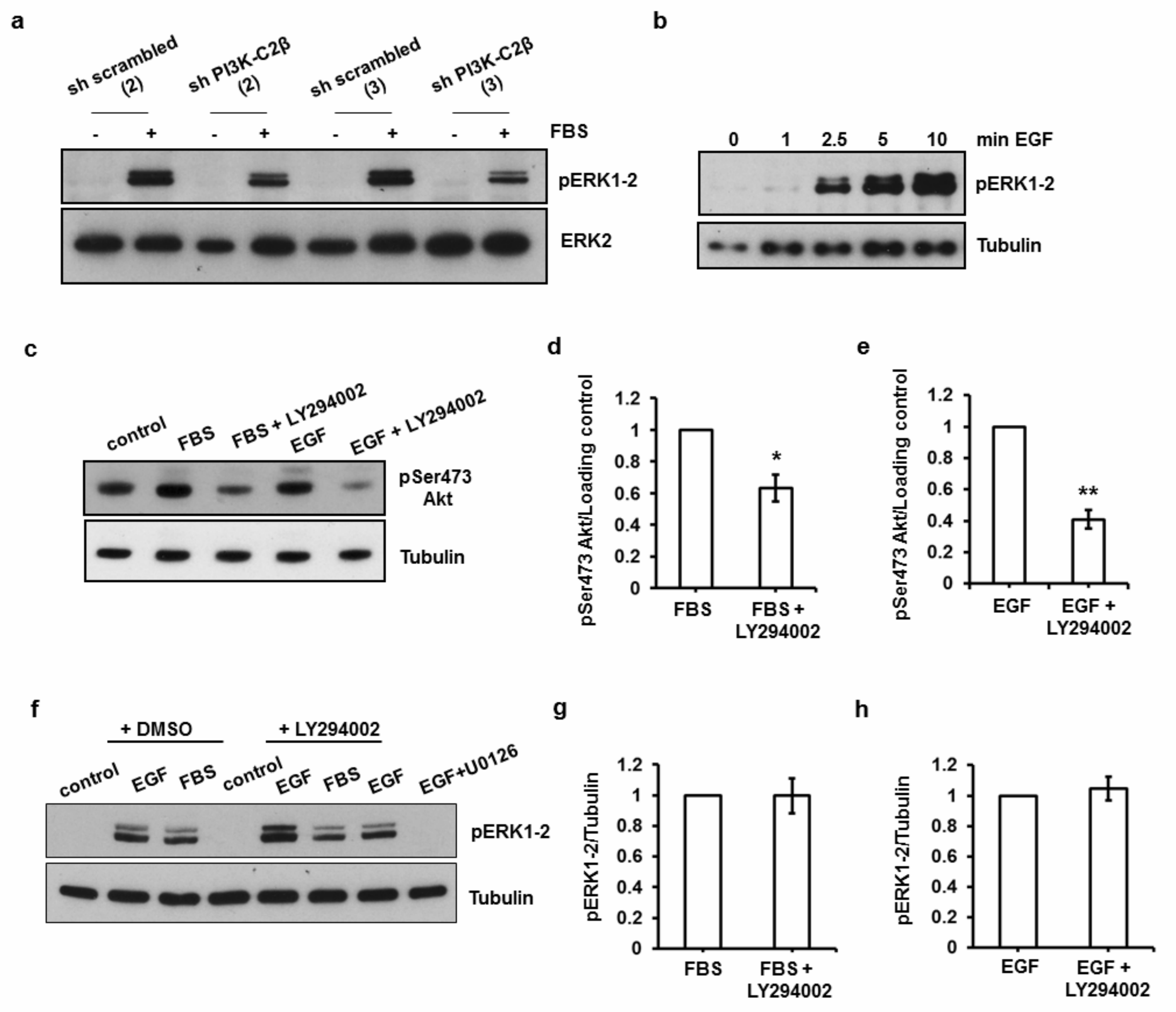

a
sh scrambled (2)
sh PI3K-C2β (2)
sh scrambled (3)
sh PI3K-C2β (3)
- + - + - + - + FBS
pERK1-2
ERK2
b
0 1 2.5 5 10 min EGF
pERK1-2
Tubulin
c
control
FBS
FBS + LY294002
EGF
EGF + LY294002
pSer473 Akt
Tubulin
d
pSer473 Akt/Loading control
FBS
FBS + LY294002
e
pSer473 Akt/Loading control
EGF
EGF + LY294002
f
+ DMSO
+ LY294002
control EGF FBS control EGF FBS EGF EGF+U0126
pERK1-2
Tubulin
g
pERK1-2/Tubulin
FBS
FBS + LY294002
h
pERK1-2/Tubulin
EGF
EGF + LY294002

Figure S3:

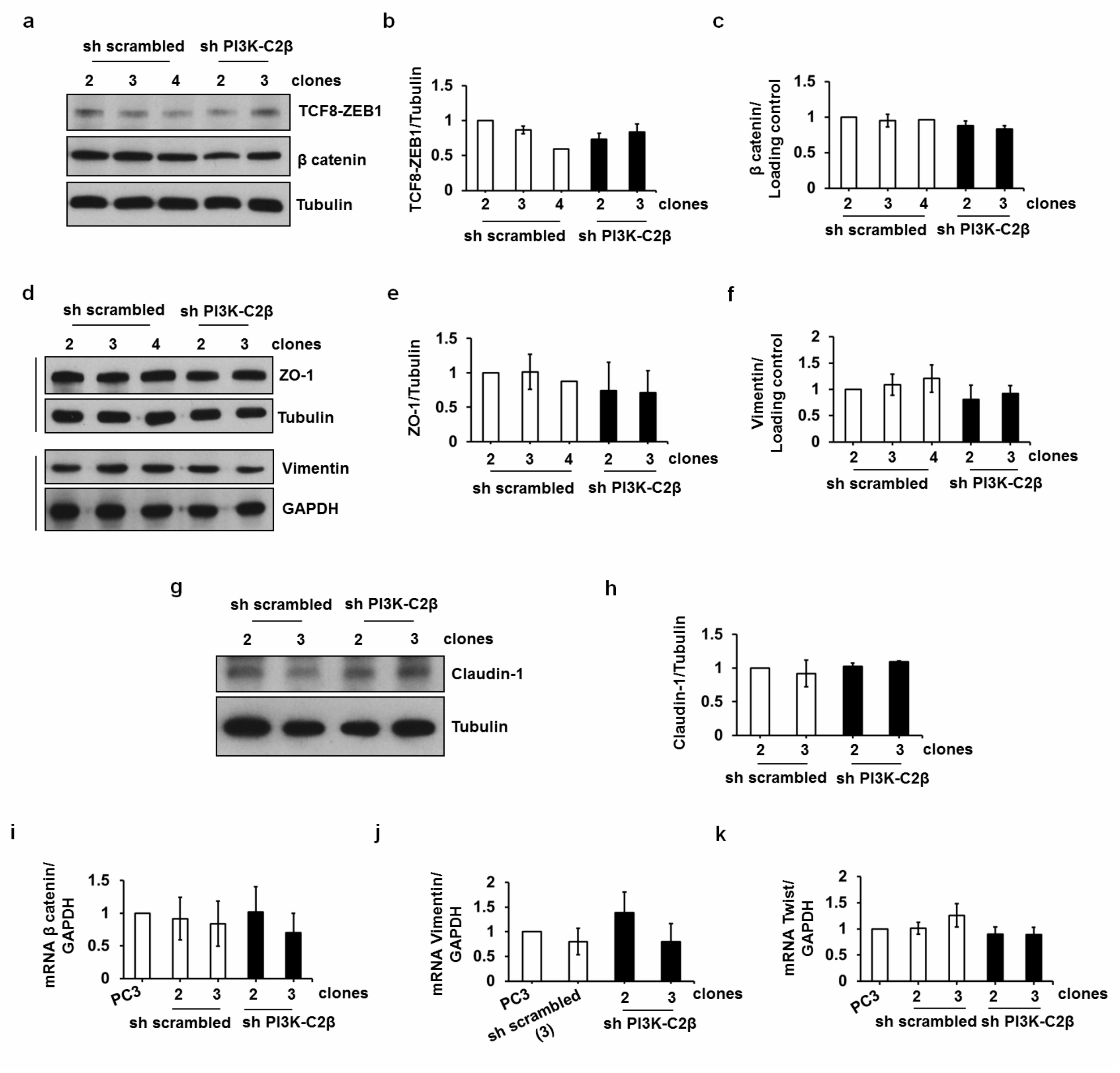
a
sh scrambled
sh PI3K-C2β
2 3 4 2 3 clones
TCF8-ZEB1
β catenin
Tubulin
b
TCF8-ZEB1/Tubulin
1.5
1
0.5
0
2 3 4 2 3 clones
sh scrambled sh PI3K-C2β
c
β catenin/
Loading control
1.5
1
0.5
0
2 3 4 2 3 clones
sh scrambled sh PI3K-C2β
d
sh scrambled
sh PI3K-C2β
2 3 4 2 3 clones
ZO-1
Tubulin
Vimentin
GAPDH
e
ZO-1/Tubulin
1.5
1
0.5
0
2 3 4 2 3 clones
sh scrambled sh PI3K-C2β
f
Vimentin/
Loading control
2
1.5
1
0.5
0
2 3 4 2 3 clones
sh scrambled sh PI3K-C2β
g
sh scrambled
sh PI3K-C2β
2 3 2 3 clones
Claudin-1
Tubulin
h
Claudin-1/Tubulin
1.5
1
0.5
0
2 3 2 3 clones
sh scrambled sh PI3K-C2β
i
mRNA β catenin/
GAPDH
1.5
1
0.5
0
PC3 2 3 2 3 clones
sh scrambled sh PI3K-C2β
j
mRNA Vimentin/
GAPDH
2
1.5
1
0.5
0
PC3 2 3 clones
sh scrambled
(3)
sh PI3K-C2β
k
mRNA Twist/
GAPDH
2
1.5
1
0.5
0
PC3 2 3 2 3 clones
sh scrambled sh PI3K-C2β
